# Supplementary material for: Targeting matrix metalloproteinase-14 disrupts DNA repair and reduces viability in adrenocortical carcinoma
Source: bioRxiv. 2026 Jan 7:2026.01.06.697992. Preprint. [Version 1] doi: 10.64898/2026.01.06.697992 (PMC12803226; doi:10.64898/2026.01.06.697992)
Supplement: Supplement 1 [file NIHPP2026.01.06.697992v1-supplement-1.pdf]

bioRxiv preprint doi: https://doi.org/10.64898/2026.01.06.697992; this version posted January 7, 2026. The copyright holder for this preprint (which was not certified by peer review) is the author/funder. This article is a US Government work. It is not subject to copyright under 17 USC 105 and is also made available for use under a CC0 license.

A

	MMP14 Median Expression					
Disease	Stage I	Stage II	Stage III	Stage IV	Stage I - IV*	p-value
THCA	5.45	5.19	5.66	5.83	0.38	**0.008**
KICH	4.33	3.81	3.83	3.74	-0.59	**0.012**
TGCT	5.15	5.7	6.44	NA	1. 29	**0.015**
ACC	3.22	3.71	4.87	5.19	1.97	**0.019**
KIRC	6.39	6.19	6.28	6.43	0.04	**0.023**
READ	5.92	6.24	6.51	6.46	N/A	0.09
COAD	6.09	6.22	6.86	6.22	N/A	0.109
CHOL	6.99	6.96	3.75	7.15	N/A	0.149
SKCM	7.93	8.17	8.01	8.4	N/A	0.21
STAD	6.47	6.78	6.66	6.81	N/A	0.222
PAAD	6.97	7.89	7.76	8.21	N/A	0.338
HNSC	7.76	7.65	7.34	7.53	N/A	0.478
ESCA	6.13	6.89	7.12	6.77	N/A	0.548
LUSC	7.04	7.19	6.92	7.85	N/A	0.553
BRCA	7.33	7.04	7.21	7.38	N/A	0.565
LIHC	3.75	4.04	4.13	4.25	N/A	0.624
UVM	NA	7.27	7.52	7.3	N/A	0.626
BLCA	6.96	6.71	7.07	7.06	N/A	0.63
KIRP	6.35	6.78	6.74	6.32	N/A	0.633
MESO	8.33	7.51	7.62	8.44	N/A	0.66
LUAD	6.34	6.43	6.65	6.23	N/A	0.888
					*For TGCT, Stage I - III	

B

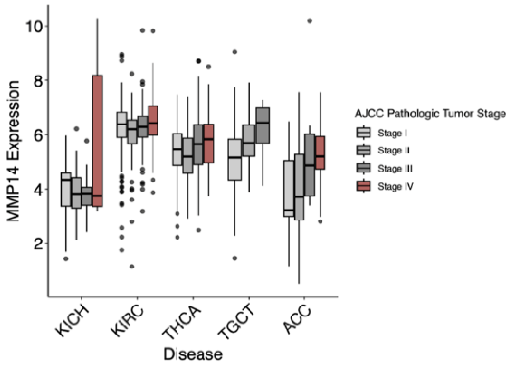

Figure S1. Pan-Cancer ANOVA Trend Analysis. A. The table demonstrating median MMP14 expression values for stage I, stage II, stage III, and stage IV cancers, stage I-IV slope, and ANOVA trend analysis p-values. B. Boxplots representing MMP14 expression in stage I-IV cancers associated with significant ANOVA trend analysis p-values.

bioRxiv preprint doi: https://doi.org/10.64898/2026.01.06.697992; this version posted January 7, 2026. The copyright holder for this preprint (which was not certified by peer review) is the author/funder. This article is a US Government work. It is not subject to copyright under 17 USC 105 and is also made available for use under a CC0 license.

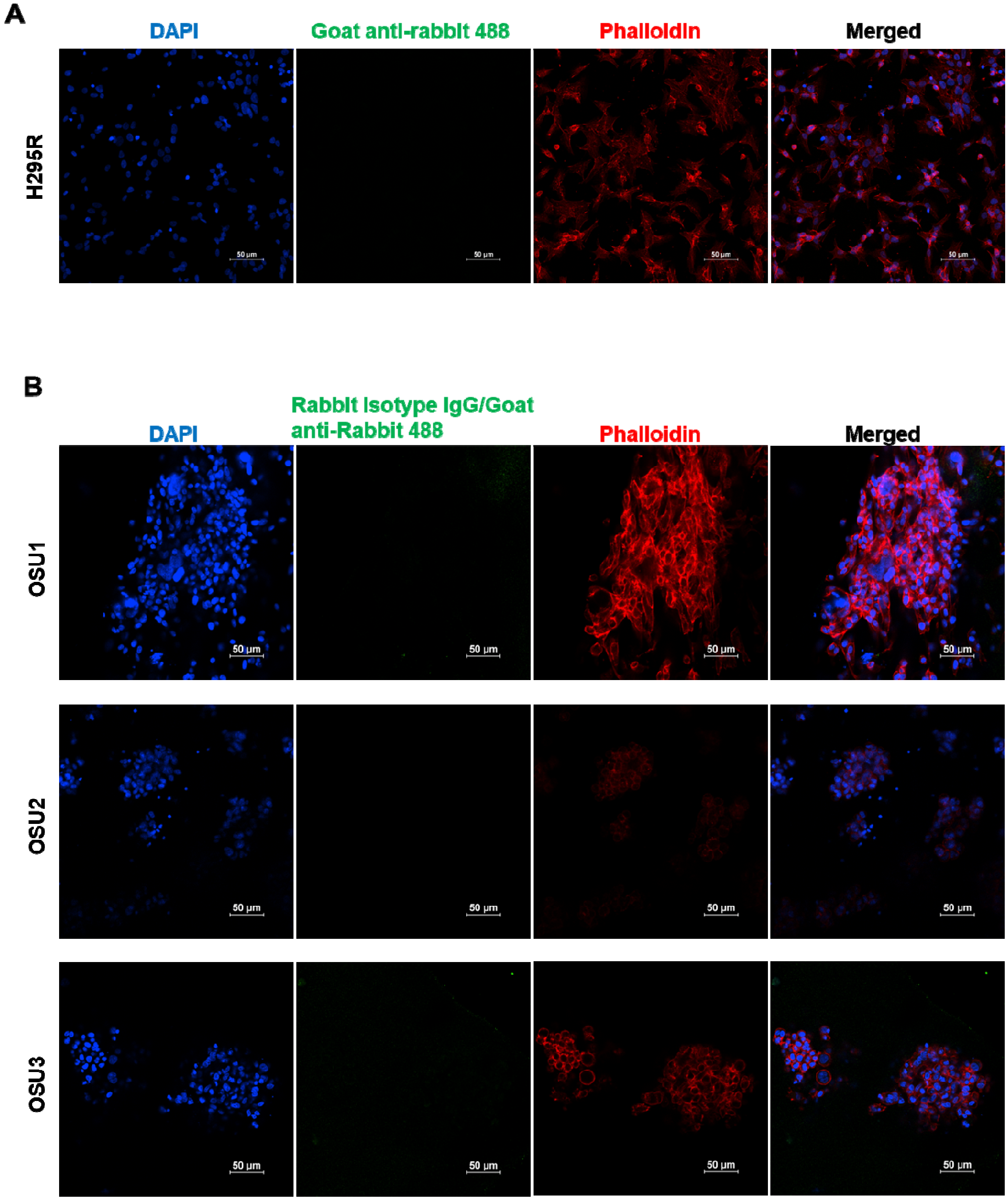

Figure S2. Isotype antibody controls for MMP-14 immunofluorescence. A. Isotype antibody controls for MMP-14 immunofluorescence in NCI-H295R cells. Related to Figure 2A. **B**. Isotype antibody controls for MMP-14 immunofluorescence in ACC PTOs. Related to Figure 3.

bioRxiv preprint doi: https://doi.org/10.64898/2026.01.06.697992; this version posted January 7, 2026. The copyright holder for this preprint (which was not certified by peer review) is the author/funder. This article is a US Government work. It is not subject to copyright under 17 USC 105 and is also made available for use under a CC0 license.

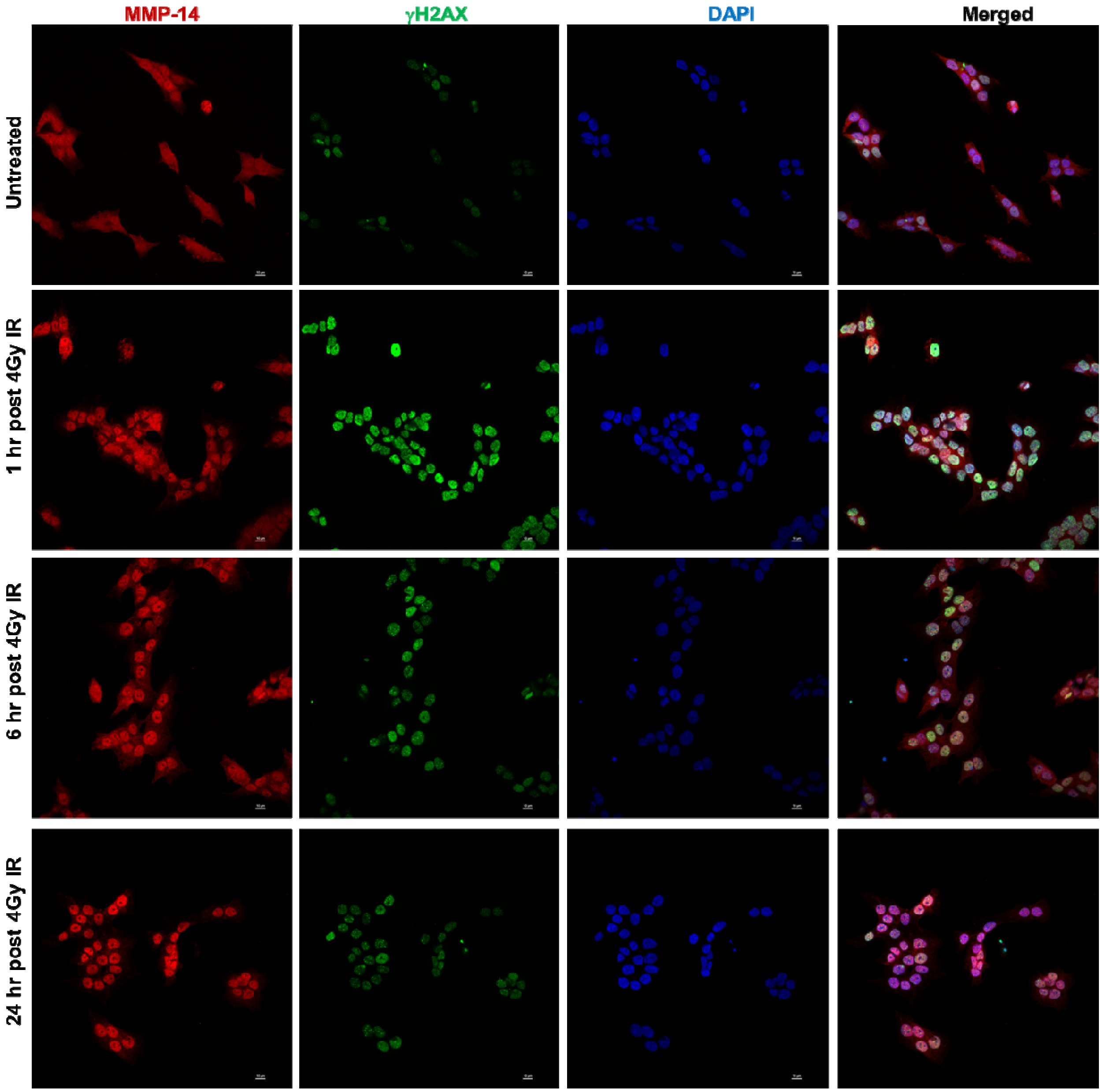

Figure S3. MMP-14 undergoes nuclear accumulation in response to ionizing radiation. Related to Figure 6B.

bioRxiv preprint doi: https://doi.org/10.64898/2026.01.06.697992; this version posted January 7, 2026. The copyright holder for this preprint (which was not certified by peer review) is the author/funder. This article is a US Government work. It is not subject to copyright under 17 USC 105 and is also made available for use under a CC0 license.

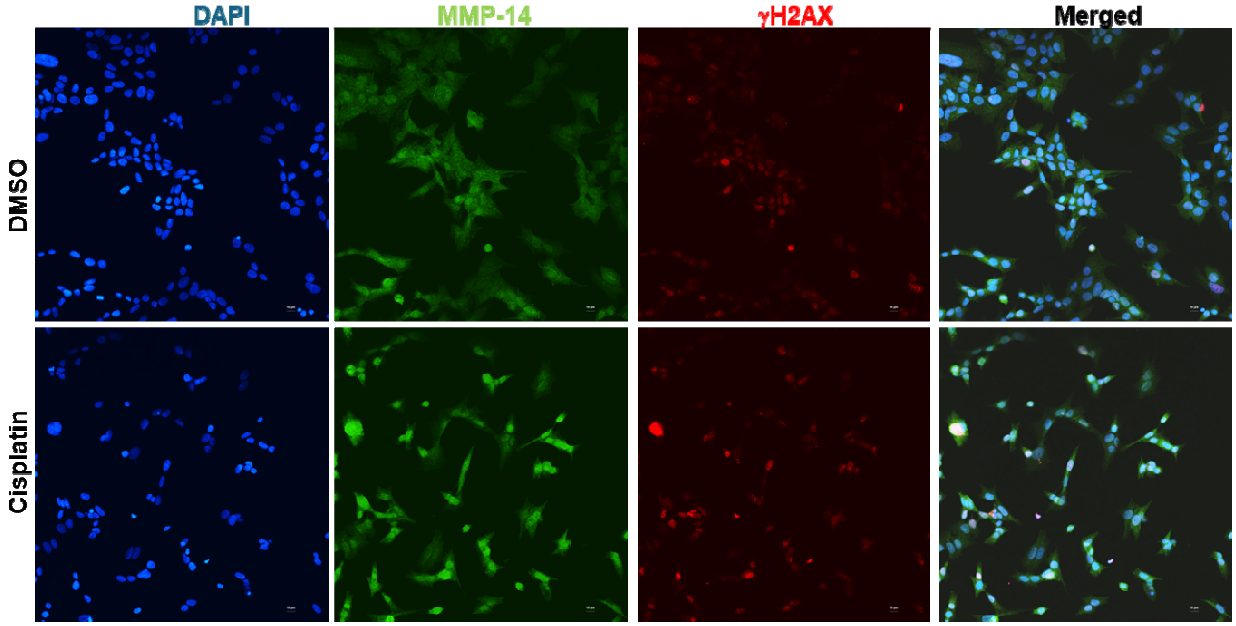

Figure S4. MMP-14 undergoes nuclear accumulation in response to cisplatin treatment. Related to Figure 6C.

bioRxiv preprint doi: https://doi.org/10.64898/2026.01.06.697992; this version posted January 7, 2026. The copyright holder for this preprint (which was not certified by peer review) is the author/funder. This article is a US Government work. It is not subject to copyright under 17 USC 105 and is also made available for use under a CC0 license.

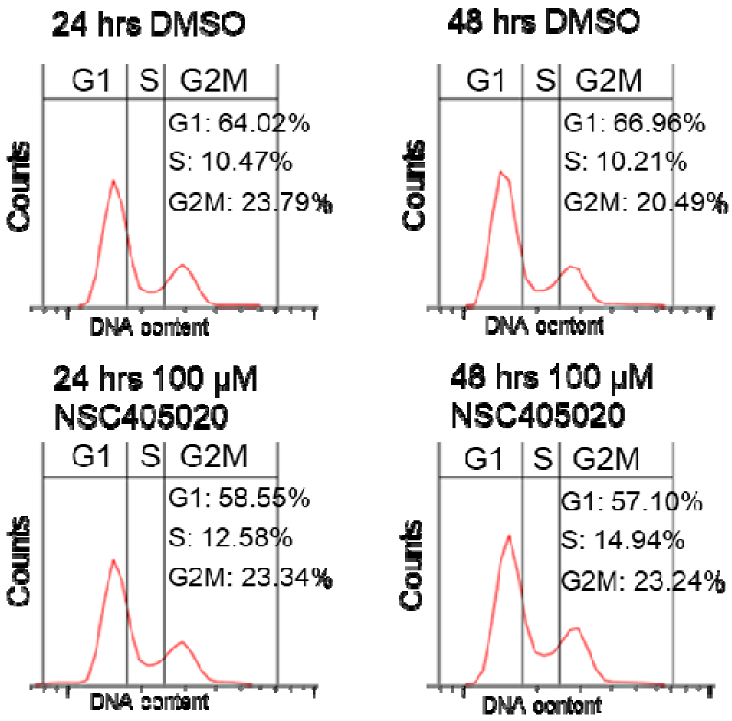

Figure S5. NSC405020 inhibition of MMP-14 does not induce S-phase cell cycle arrest.

Related to Figure 5.
